# Clinical relevance of SCN and CyN induced by ELANE mutations: a systematic review

**DOI:** 10.3389/fimmu.2024.1349919

**Published:** 2024-05-22

**Authors:** Yufan Xiao, Nandi Wang, Xinghao Jin, Anna Liu, Zhiyong Zhang

**Affiliations:** ^1^ Department of Rheumatology and Immunology, Children’s Hospital of Chongqing Medical University, Chongqing, China; ^2^ Department of Pediatrics, West China Second University Hospital, Sichuan University, Chengdu, China; ^3^ Ministry of Education Key Laboratory of Child Development and Disorders, Children’s Hospital of Chongqing Medical University, Chongqing, China; ^4^ National Clinical Research Center for Child Health and Disorders, Children’s Hospital of Chongqing Medical University, Chongqing, China; ^5^ China International Science and Technology Cooperation Base of Child Development and Critical Disorders, Children’s Hospital of Chongqing Medical University, Chongqing, China; ^6^ Chongqing Key Laboratory of Child Infection and Immunity, Children’s Hospital of Chongqing Medical University, Chongqing, China

**Keywords:** congenital neutropenia, cyclic congenital neutropenia, ELANE, severe congenital neutropenia, primary immunodeficiency disease

## Abstract

**Introduction:**

According to the PRISMA criteria, a systematic review has been conducted to investigate the clinical relevance between patients with severe congenital neutropenia (SCN) and cyclic congenital neutropenia (CyN) induced by ELANE mutations.

**Methods:**

We have searched PubMed, EMBASE, Web of Science, Scopus, Cochrane, CNKI, Wanfang Medicine, and VIP for ELANE mutation related literature published from 1997 to 2022. Using Microsoft Excel collect and organize data, SPSS 25, GraphPad Prism 8.0.1, and Omap analyze and plot statistical. Compare the gender, age, geography, mutation sites, infection characteristics, treatment, and other factors of SCN and CyN patients induced by ELANE mutations, with a focus on exploring the relationship between genotype and clinical characteristics, genotype and prognosis.

**Results:**

This study has included a total of 467 patients with SCN and 90 patients with CyN. The onset age of SCN and CyN are both less than 1 year old, and the onset and diagnosis age of SCN are both younger than CyN. The mutation of ELANE gene is mainly missense mutation, and hot spot mutations include S126L, P139L, G214R, c.597+1G>A. The high-frequency mutations with severe outcomes are A57V, L121H, L121P, c.597+1G>A, c.597+1G>T, S126L, C151Y, C151S, G214R, C223X. Respiratory tract, skin and mucosa are the most common infection sites, Staphylococcus aureus, Pseudomonas aeruginosa and Escherichia coli are the most common.

**Discussion:**

Patients with refractory G-CSF are more likely to develop severe outcomes. The commonly used pre-treatment schemes for transplantation are Bu-Cy-ATG and Flu-Bu-ATG. The prognosis of transplantation is mostly good, but the risk of GVHD is high.

**Systematic Review Registration:**

https://www.crd.york.ac.uk/PROSPERO/. PROSPERO, identifier CRD42023434656.

## 1 Introduction

The most common cause of severe congenital neutropenia (SCN) is the mutation of the neutrophil elastase gene (*ELANE*), which can also lead to another rare autosomal dominant genetic disorder, namely, cyclic congenital neutropenia (CyN). The characteristics of both are continuous or periodic changes in absolute neutrophil count (ANC). Patients manifest recurrent infections in infancy, often developing more severe SCN (1, 2). SCN, considered a precancerous state, was first identified as preleukemia syndrome in 1970 (3), which has the risk of malignant transformation to myelodysplastic syndrome (MDS) or acute myeloid leukemia (AML). It has been reported that CyN has no risk of MDS/AML, and the biological basis of the significant difference between CyN and SCN in malignant transformation is still unclear (4–6). Continuous blood routine monitoring and bone marrow cytology are important tests for the diagnosis of SCN and CyN caused by *ELANE* mutation, and genetic testing is the gold standard for diagnosis. In addition to prophylactic and anti-infective therapy, granulocyte colony-stimulating factor 3 receptor (G-CSF) is also used as a first-line therapy (7), but there are currently no accepted guidelines for its use. Hematopoietic stem cell transplantation (HSCT) is a curative option, but the timing of transplantation remains controversial (2, 8).

In summary, the study intends to systematically review the clinical manifestations, *ELANE* mutation characteristics, G-CSF treatment, and HSCT transplantation between SCN and CyN induced by *ELANE* mutation so as to provide a reference for scientific and standardized decision-making in clinical diagnosis and treatment and help further mechanism research.

## 2 Methods

**Figure 1:**
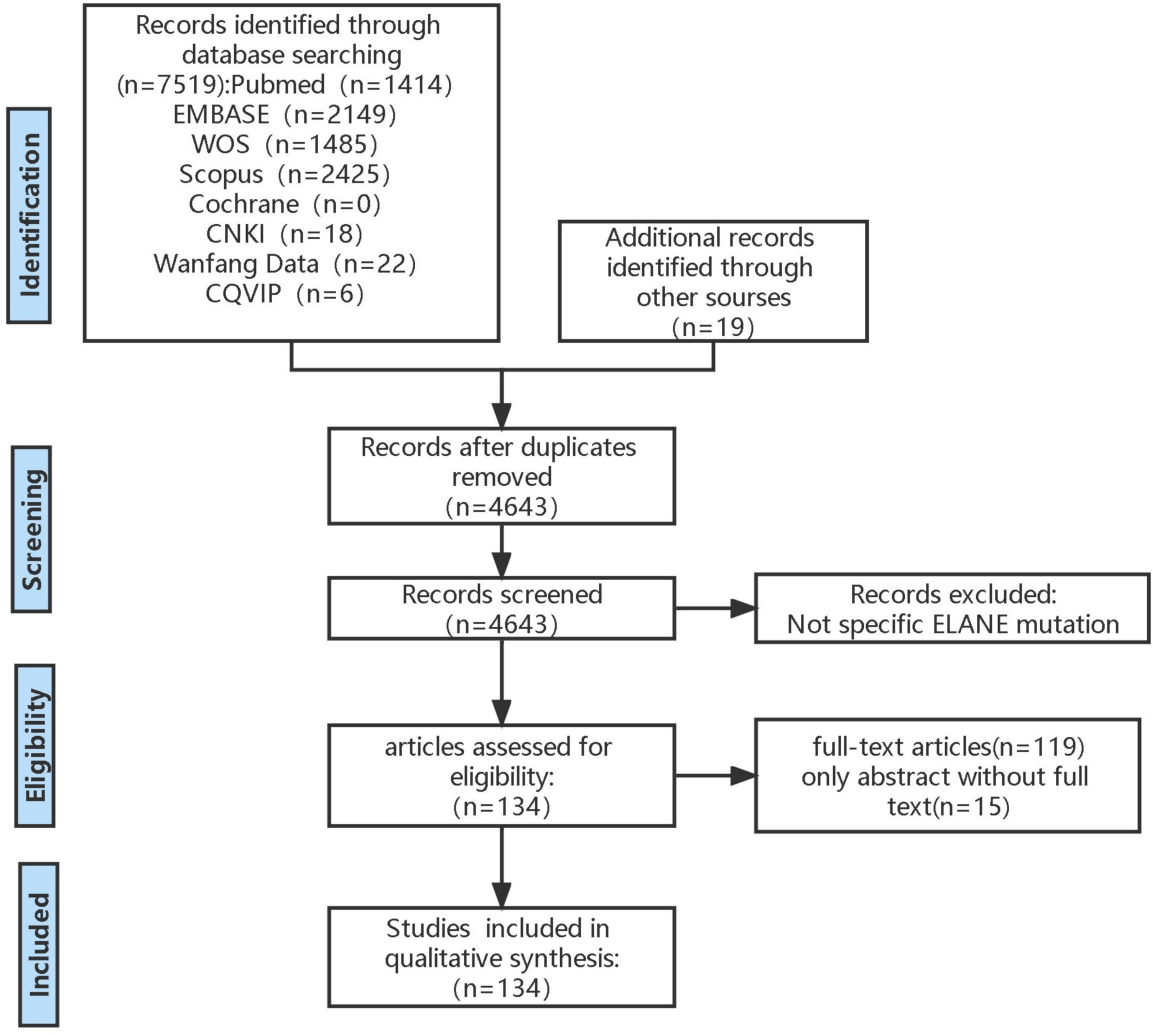
PRISMA flow diagram. This is inclusion and exclusion flowchart used PRISMA criteria. PRISMA, Preferred Reporting Items for Systematic Reviews and Meta-Analyses.

This systematic review was conducted in accordance with Preferred Reporting Items for Systematic Reviews and Meta-Analyses (PRISMA) criteria (PROSPERO registration number: CRD42023434656) (**Figure 1**).

### 2.1 Literature search

Eight databases were searched—PubMed, EMBASE, Web of Science, Scopus, Cochrane, CNKI, Wanfang Medicine, and VIP—for *ELANE* mutation-related literature published from 1997 to 2022, including but not limited to reviews, case reports, and clinical studies. The key search terms were keywords containing “*ELANE*” or “congenital neutropenia” or title/abstract containing “*ELANE*” and “neutropenia” and keywords containing “severe congenital neutropenia” or “cyclic neutropenia” or “SCN” or “CyN” or “CN” or “primary immunodeficiency disease” or “PID”. In some cases, the corresponding authors of these articles were contacted. The information of patients treated at the Children’s Hospital of Chongqing Medical University included the basic information, clinical features, *ELANE* mutation sites, treatment, and other information of patients through the hospital’s electronic medical record system.

### 2.2 Inclusion and exclusion criteria

After duplicate articles were excluded, abstracts and full texts were screened. Studies and case reports must describe specific *ELANE* mutation sites to be eligible for inclusion. Patients with other immunodeficiency diseases, connective tissue diseases, rheumatism, and hematologic tumor diseases other than congenital neutropenia were excluded.

### 2.3 Quality assessment

This retrospective review has some limitations. Some information about each manifestation could not be collected. Patients were been treated and evaluated by different clinicians, leading to incomplete clinical features. This article conducted a retrospective study of literature and cases. There was admission rate bias and reporting bias, as the included published patients had only limited medical information, and the information was selectively disclosed. Because the patient review information is incomplete, recall bias, lack of accuracy or completeness in the collected data, and systematic errors with the real situation. Due to the researcher’s prior understanding of the disease situation and outcome, exploring factors related to the outcome resulted in exposure suspicion bias.

### 2.4 Data extraction

Researchers (Y.F. Xiao and N.D. Wang) used the above method to search the literature library separately, screened the literature based on inclusion and exclusion criteria, and collected data using standardized tables. The standard form includes collected data such as gender, age of onset, age of diagnosis, positive or sporadic family history, gene mutation site, mutation type, clinical characteristics, serious outcomes, G-CSF treatment, and HSCT. If there were any disagreements during the process, other researchers were requested to make a decision.

### 2.5 Statistical analysis

Data were collected and organized using Microsoft Excel (Microsoft), and statistical analysis was performed using SPSS 25. The graph software GraphPad Prism 8.0.1 and Omap were used. The distribution trend was described using (number of cases and percentage) for sample rates, the chi-square test was used for comparison of sample rates, and the correlation coefficient of Pearson or Spearman was used to analyze the correlation. Since the measurement data mentioned in this study were all skewed distributions, the median (minimum–maximum) and mode were used to describe the distribution trend. p < 0.05 was considered statistically significant with the significance level α = 0.05.

## 3 Results

### 3.1 Baseline characteristics of SCN and CyN patients

This study includes a total of 134 articles related to *ELANE* mutations published from 1997 to 2022 divided into 467 SCN patients and 90 CyN patients.

The sex ratio of SCN patients was 1.10 (130 male and 118 female patients), and that of CyN patients was 0.81 (22 male and 27 female patients). The onset age was analyzed using a one-sample t-test. The test value was set to 1 year old, and the onset age of the two was statistically significant less than 1 year (**Table 1**). There were 55 patients (55/69, 79.71%) with SCN with an onset age of less than 1 year and eight patients (8/12, 66.67%) with CyN with an onset age of less than 1 year. The distribution trend of onset age was as follows (**Table 1**): the median onset age of SCN was 0.16 years (0–3.00 years), and the median onset age of CyN was 0.53 years (0.08–3.00 years). Taking the time of genetic diagnosis as the age of diagnosis (**Table 1**), the median age of diagnosis for SCN patients was 1.50 years (0.00–39.00 years), and the median age of diagnosis for CyN patients was 7.00 years (0.08–28.00 years).

**Table 1:** Onset age and diagnosis age of patients with *ELANE* mutation.

	Onset age		Diagnosis age	
	SCN	CyN	SCN	CyN
Cases	69	12	180	27
Average	0.48	0.92	4.55	9.12
Minimum	0.00	0.08	0.00	0.08
Maximum	3.00	3.00	39.00	28.00
Median	0.16	0.53	1.50	7.00

SCN, severe congenital neutropenia; CyN, cyclic congenital neutropenia.

According to the analysis of the hospital location at the time of diagnosis, SCN patients were mainly distributed in the United States (93/429, 21.68%), France (76/429, 17.72%), and China (56/429, 13.05%); CyN patients were mainly distributed in France (37/89, 43.82%), Germany (9/89, 10.11%), and China (9/89, 10.11%).

### 3.2 Clinical features of SCN and CyN patients

**Figure 2:**
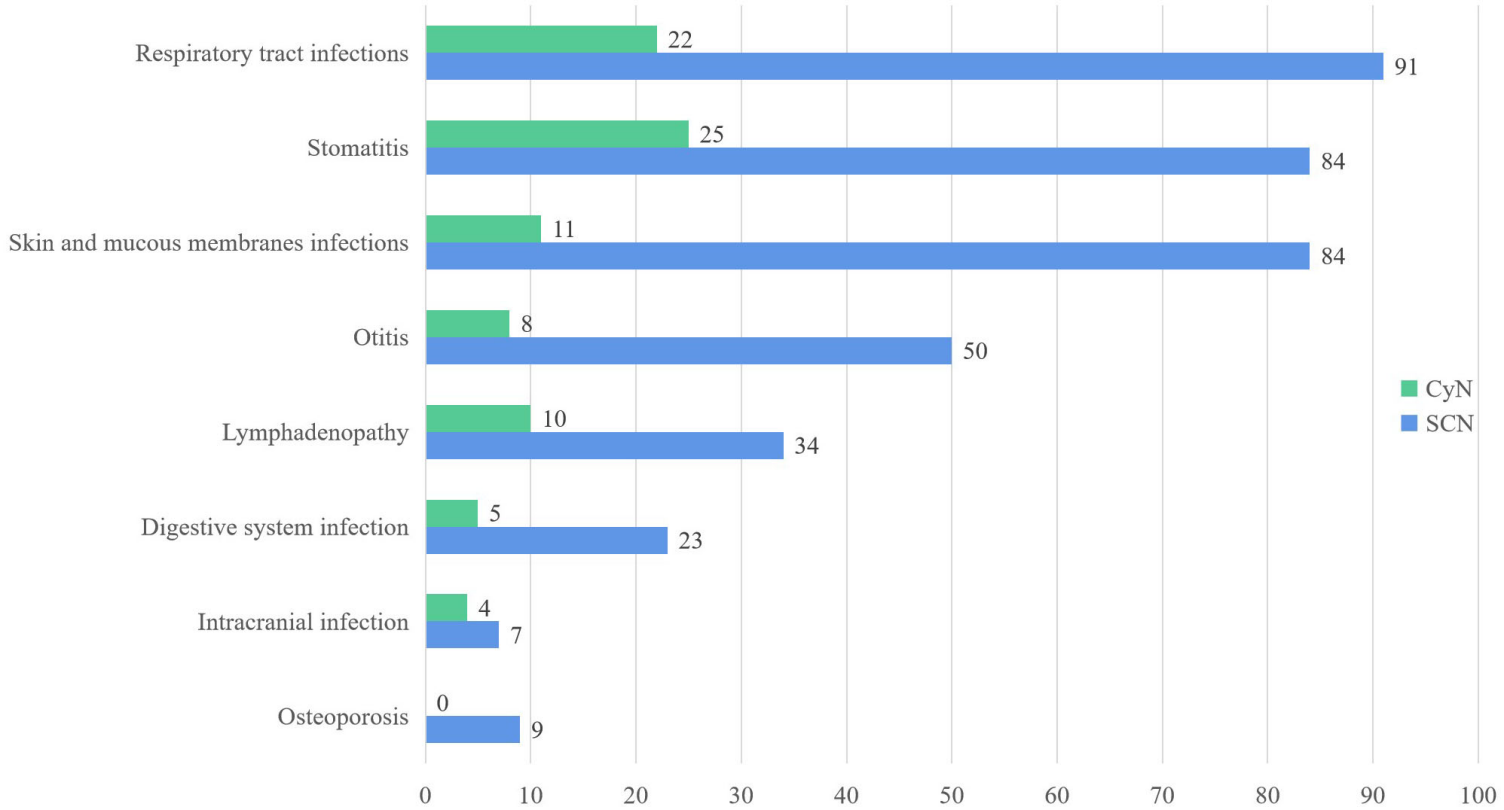
Clinical features of *ELANE* gene mutations in SCN and CyN patients. SCN, severe congenital neutropenia; CyN, cyclic congenital neutropenia.

Both SCN and CyN patients were characterized by recurrent respiratory system infections (SCN 91/235, 38.72%; CyN 22/45, 48.89%) and skin mucosal infections (SCN 84/235, 35.74%; CyN 11/45, 24.44%), especially stomatitis (SCN 84/235, 35.74%; CyN 25/45, 55.56%). Considering that the same patient can experience multiple infections and infections in different parts, the frequency of clinical symptoms appearing in the literature was statistically analyzed (**Figure 2**).

Periodontitis and aphthous stomatitis were the most common forms of stomatitis. Periodontitis includes gingivitis, loose teeth, swollen gums, bleeding gums, alveolar bone resorption, and other manifestations. In addition, there were caries, alveolar bone resorption, fungal stomatitis, and other manifestations. Respiratory tract infection was manifested as upper respiratory tract infection, bronchitis, pneumonia, lung consolidation, pleurisy/pleural effusion, lung abscess, respiratory failure, tracheomalacia, etc. Upper respiratory tract infection was divided into rhinitis, pharyngitis, and tonsillitis. The main site of skin mucosal infection was the umbilical and perianal regions, with the form of abscesses and cellulitis. In addition, *ELANE* mutant SCN and CyN also often present symptoms such as otitis media, diarrhea, liver abscess, intracranial infection, anemia, and bone loss.

The risk of serious outcomes in SCN was higher, such as MDS (25/235, 10.64%), AML (49/235, 20.85%), acute lymphocytic leukemia (ALL) (5/235, 2.13%), and death (15/235, 6.38%). Only one case of CyN reported malignant transformation to AML (9), and only one CyN case of *ELANE* mutation died (1). There was a significant difference in the frequency of serious outcomes between SCN and CyN.

**Figure 3:**
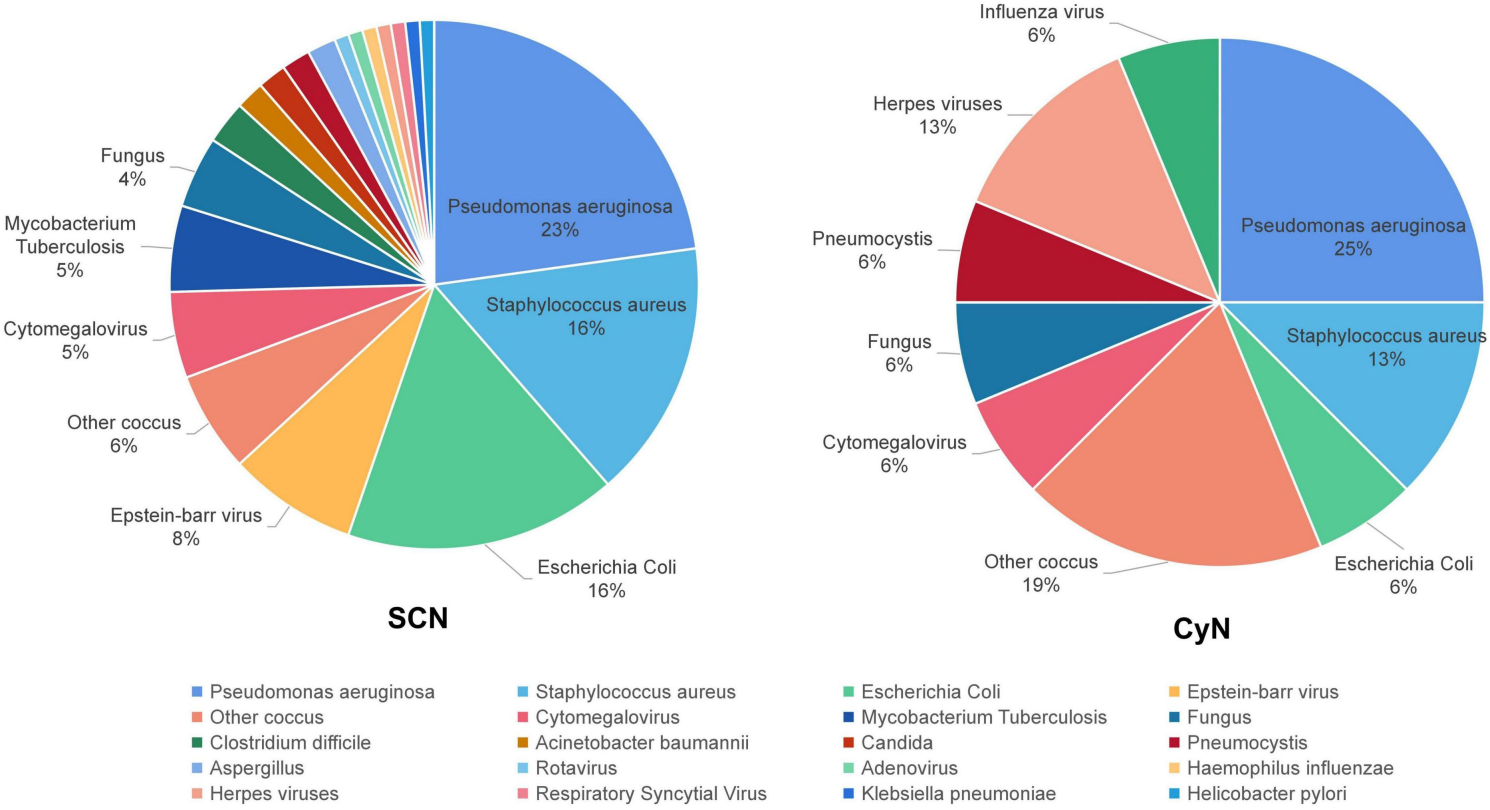
Pathogen distribution of *ELANE* gene mutations in SCN and CyN patients. SCN, severe congenital neutropenia; CyN, cyclic congenital neutropenia.

The same patient can have multiple pathogen infections. Among them, 30 SCN patients and four CyN patients had at least two pathogen infections. Sixty-three SCN patients were detected to have 114 positive pathogens, and 11 CyN patients were detected to have 16 positive pathogens: bacteria (SCN 84/114, 73.68%; CyN 10/16, 62.50%), viruses (19/114, 16.67%; 4/16, 25.00%), fungi (SCN 10/114, 8.77%; CyN 2/16, 12.50%), and parasites (SCN 1/114, 0.88%) (**Figure 3**). Bacterial infection were divided into *Pseudomonas aeruginosa* (SCN 26/114, 22.81%; CyN 3/16, 18.75%), *Escherichia coli* (SCN 19/114, 16.67%; CyN 1/16, 6.25%), *Staphylococcus aureus* (SCN 18/114, 15.79%; CyN 2/16, 12.50%), *Mycobacterium tuberculosis* (SCN 6/114, 5.26%), *Clostridium difficile* (SCN 3/114, 2.63%), *Acinetobacter baumannii* (SCN 2/114, 1.75%), *Haemophilus influenzae* (SCN 1/114, 0.88%), *Helicobacter pylori* (SCN 1/114, 0.88%), *Klebsiella pneumoniae* (SCN 1/114, 0.88%), *P. aeruginosa* (CyN 1/16, 6.25%), other cocci (SCN 7/114, 6.14%; CyN 3/16, 18.75%). Viral infections were divided into Epstein–Barr (EB) virus (SCN 9/114 7.89%), *Cytomegalovirus* (SCN 6/114 5.26%; CyN 1/16, 6.25%), herpes virus (SCN 1/114, 0.88%; CyN 2/16, 12.56%), influenza virus (CyN 1/16, 6.25%), respiratory syncytial virus (RSV) (SCN 1/114, 0.88%), *Rotavirus* (SCN 1/114, 0.88%), and *Adenovirus* (SCN 1/114, 0.88%). Fungal infections were divided into *Candida* (SCN 2/114, 1.75%; CyN 1/16, 6.25%), *Aspergillus* (SCN 2/114, 1.75%), and other fungi (SCN 5/114, 4.38%; CyN 1/16, 6.25%). Only one case of pulmonary cysticercosis infection was reported in SCN patients with parasites (10).

### 3.3 Mutation characteristics

**Figure 4:**
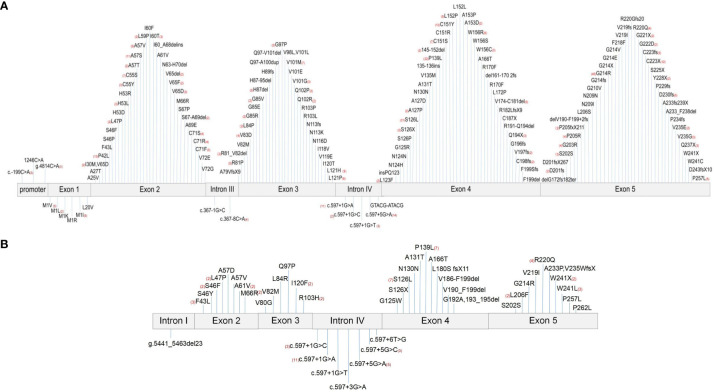
Distribution map of *ELANE* gene mutations. **(A)** Distribution map of *ELANE* gene mutations in SCN patients. **(B)** Distribution map of *ELANE* gene mutations in CyN patients. SCN, severe congenital neutropenia; CyN, cyclic congenital neutropenia.

*ELANE* is located on chromosome 19p13.3 and contains five exons and six introns. This study collected 161 different *ELANE* mutation sites from SCN patients and 43 different sites from CyN patients. There were seven identical *ELANE* mutation sites in SCN and CyN, resulting in a total of 197 different *ELANE* mutation sites. SCN mutation sites were mainly distributed in Exon 2, Exon 3, Exon 4, and Exon 5. S126L (31/467, 6.64%), P139L (22/467, 4.71%), and G214R (42/467, 8.99%) were hotspot mutations (**Figure 4A**); CyN mutation sites were mainly distributed in Intron IV, Exon 4, and Exon 5, with c.597 + 1G>A (11/90, 12.22%), S126L (7/90, 7.78%), and P139L (7/90, 7.78%) as hotspot mutations (**Figure 4B**).

The main types of mutations were missense mutation (SCN 314/439, 72.67%; CyN 55/91, 60.44%), and the rest were cases of nonsense mutation (SCN 29/439, 6.61%; CyN 2/91, 2.20%), frameshift mutations (SCN 45/439, 10.25%; CyN 6/91, 6.59%), synonymous mutations (SCN 9/439, 2.05%; CyN 2/91, 2.20%), and intron mutations (SCN 42/439, 9.57%; CyN 26/91, 28.57%).

### 3.4 Clinical features in relation to gene mutations

This study explores the relationship between clinical features and gene mutations from three aspects: 1) the same genotype exhibiting different disease phenotypes, 2) a comparison of clinical manifestations among different members of the family, and 3) the relationship between *ELANE* genotype and poor prognosis.

There were reports of both SCN- and CyN-related mutations in the *ELANE* gene, including A57V, G97P, S126L, P139L, W241X, c.597 + 1G>A, and c.597 + 5G>A (**Table 2**). In other words, there were two different disease phenotypes at the same mutation site, with its pathogenesis worthy of further study.

**Table 2:** Summary of phenotypes of different diseases of the same *ELANE* mutation.

	Nucleotide (NM_001972.4)	Protein (NP_001963.1)	SCN (n = 84)	CyN (n = 33)
Exon 2	c.170C>T	A57V	5	1
Exon 3	c.290A>C	G97P	2	1
Exon 4	c.377C>T	S126L	28	7
Exon 4	c.416C>T	P139L	21	7
Intron IV	c.597 + 1G>A	/	11	10
Intron IV	c.597 + 5G>A	/	12	5
Exon 5	c.723G>A	W241X	1	2

SCN, severe congenital neutropenia; CyN, cyclic congenital neutropenia.

In this study, 123 cases with clear family history information (immediate relatives with complete ELANE genetic testing) were collected, including 66 cases with positive family history: 45 cases of SCN (45/89, 50.56%) and 21 cases of CyN (21/34, 61.76%). Two typical family cases were collected. First, 10 children had the same paternal origin of *ELANE* gene mutation S126L (all mothers had no *ELANE* gene mutation), of which two children were asymptomatic, seven children showed SCN, and one child showed CyN (11, 12). Second, a Korean woman (c.597 + 1G>A) had three daughters, with the mother only having recurrent stomatitis and two of her daughters both exhibiting SCN with c.597 + 1G>A *ELANE* mutation (13). One had recurrent respiratory infections. However, the other one showed severe infection, with life-threatening peritonitis, sepsis, and severe pneumonia. Family members carrying the same *ELANE* mutation exhibited completely different clinical phenotypes, indicating that the complex relationship between the *ELANE* gene and clinical phenotype needs further research.

This article summarizes the mutation sites of the *ELANE* gene sites in patients with serious outcomes (**Table 3**), including A57V, L121H, L121P, c.597 + 1G>A, c.597 + 1G>T, S126L, C151Y, C151S, G214R, and C223X, and all 10 high-frequency mutation sites were prone to developing AML. The related sites of MDS were L121P, c.597 + 1G>T, S126L, C151Y, C151S, and G214R, and the related sites of acute lymphoblastic leukemia were A57V and G214R. There was a total of 16 cases of G214R with serious outcomes, and the probable outcomes were MDS (4/43, 9.30%), AML (9/43, 20.93%), ALL (2/43, 4.65%), and death (1/43, 2.33%). Although HSCT treatment is influenced by multiple factors such as clinical symptoms, donors, and economy, patients carrying G214R (13/43, 30.23%) and L121H (5/8, 62.50%) had a high frequency of transplantation treatment, which is worthy of attention.

**Table 3:** Summary of genotypes and serious outcomes of *ELANE* mutation.

Outcome	Exon 2	Intron III	Exon 3	Intron IV	Exon 4	Exon 5
**MDS**	H53L V65D	/	V82M H87del L121P (2)	c.597 + 1G>T c.597 + 5G>A (2)	S126L C151Y (3) C151S (4) W156C	G214R (4) D230fs Q237X
**AML**	H53L A57V (2) I60T	c.367–8C>A	V82M H87del V101M N113K L121H L121P	c.597 + 1G>A c.597 + 1G>T	S126X S126L C151Y (6) C151S L152P (2)	S202S G203R G214R (9) C223X (5) Y228X D230fs Q237X D243fsX10
**ALL**	A57V (2)	/	L121H	/	V174_C181del	G203R G214R (2)
**Death**	S67P V65del (2)	/	/	c.597 + 5G>A	S126L 135–136ins5 C151S W156R (2) Q194X (2)	P205R G214R V219I C223X

The red numbers in parentheses represent the number of cases.

MDS, myelodysplastic syndrome; AML, acute myeloid leukemia; ALL, acute lymphocytic leukemia.

### 3.5 Treatment and outcomes

Information on the use of G-CSF was collected from 150 cases of SCN and 21 cases of CyN. The distribution trends of initial dose, maximum dose, and minimum dose in CyN patients were 2.00 ± 3.17 μg/(kg·d), 5.00 ± 40.00 μg/(kg·d), and 2.00 ± 3.00 μg/(kg·d), respectively. The distribution trends of initial dose, maximum dose, and minimum dose in SCN patients were 6.00 ± 4.00 μg/(kg·d), 20.00 ± 30.00 μg/(kg·d), and 6.00 ± 4.00 μg/(kg·d), respectively. The dosage of G-CSF ≥ 5 μg/(kg·d) was defined as a large dosage, and the data of 114 patients were collected (103 SCN patients and 11 CyN patients), of which 45 patients developed serious outcomes. Compared with 38 patients treated with G-CSF <5 μg/(kg·d), a large dosage of G-CSF was more likely to result in serious outcomes as analyzed using the chi-square test.

The average transplant age of 43 patients was 5.65 years, and 75% of patients underwent HSCT before the age of 6.50. The source of transplant donors was as follows: related donors (7/31, 23.00%) and unrelated donors (24/31, 77.00%), providing cord blood stem cells (10/25, 40.00%), bone marrow hematopoietic stem cells (9/25, 36.00%), and peripheral hematopoietic stem cells (6/25, 24.00%) for transplantation. A total of 19 pre-treatment schemes were collected, with the most common being busulfan–cyclophosphamide–anti-human thymocyte immunoglobulin (Bu-Cy-ATG) (18/58, 31.03%) and fludarabine–busulfan–anti-human thymocyte immunoglobulin (Flu-Bu-ATG) (7/58, 12.07%). After transplantation, 24 cases (24/43, 55.81%) were successful, with nine patients experiencing varying degrees of graft-versus-host disease (GVHD) (9/43, 20.93%), and three patients ultimately died (3/43, 6.98%). The causes of death were infection in one case and GVHD in two cases. The hotspot mutation sites mentioned above were counted for transplant events, and the transplant proportions were G214R (16/43, 37.21%), S126L (5/38, 13.16%), P139L (1/29, 3.45%), and c.597 + 1G>A (2/22, 9.09%).

## 4 Discussion

According to the different changes in absolute neutrophil count and clinical symptoms, the phenotypes of *ELANE* mutations can be divided into SCN and CyN. According to this study, the number of SCN patients was much higher than that of CyN patients (467:90). The onset age of both SCN and CyN was less than 1 year. Additionally, CyN patients were older than SCN patients in terms of age of onset and diagnosis.

Both SCN and CyN exhibited recurrent respiratory tract infections and skin mucosal infections. Stomatitis had the highest incidence rate in respiratory tract infections; at the same time, periodontitis and aphthous stomatitis were the most common. Skin and mucosal infections mainly occurred in the umbilicus and perianal region, often manifested as abscesses and cellulitis. In addition, SCN and CyN also often showed symptoms such as otitis media, diarrhea, liver abscess, intracranial infection, anemia, and bone loss. Patients with SCN and CyN were mainly infected by bacteria, the most common being *P. aeruginosa*, *S. aureus*, and *E. coli*.

SCN can lead to serious outcomes, such as MDS, AML, ALL, and even death (5, 14, 15). It is generally believed that the malignant transformation rate of CyN patients with *ELANE* mutation is almost zero (6). However, in 2016, a case of CyN malignant transformation into acute leukemia was reported, which was a female patient with A233P and V235WfsX and finally developed into acute myeloid leukemia at the age of 17, requiring hematopoietic stem cell transplantation (9). In addition, one CyN patient with simple *ELANE* mutation F43L died (1). The specific cause of death was not mentioned, but the number of neutrophils in this patient did not increase after treatment with G-CSF. As mentioned above, CyN may have pulmonary consolidation, respiratory failure, impetigo, meningitis, infectious shock, etc. (16–18) Although the overall clinical symptoms of CyN were not as severe as those of SCN, they should still be taken seriously by clinical doctors.

The mutation sites of SCN were mainly distributed in Exon 2, Exon 3, Exon 4, and Exon 5. S126L, P139L, and G214R were high-frequency hotspot mutations. The mutation sites of CyN were mainly distributed in Intron IV, Exon 4, and Exon 5. The hotspot mutations were c.597 + 1G>A, S126L, and P139L. The main mutation type was a missense mutation. According to the information collected in this study, synonymous mutations (SCN 2.05%; CyN 2.20%) have been reported in both SCN and CyN, but the pathogenesis of synonymous mutations has not been further elucidated in relevant literature (19–22). The speculated reasons are as follows: first, due to the long time span of the literature collected in this study, before 2005, Sanger sequencing was the main method, only targeting single gene mutations, and it cannot be ruled out that the pathogenic genes were not detected. Second, the pathogenic factor may be that synonymous mutations may affect other biological processes, such as transcription factor recognition, mRNA splicing, folding, and degradation, as well as the initiation, efficiency, and accuracy of protein translation (23).

Based on the collected genotypes and clinical features, this study attempted to explore the relationship between the two from the following three aspects: 1) the same genotype exhibits different disease phenotypes, such as A57V, G97P, S126L, P139L, W241X, c.597 + 1G>A, and c.597 + 5G>A. These seven *ELANE* mutation sites can be manifested as SCN or CyN. 2) Comparison of clinical manifestations among one family: as mentioned in the two typical family cases (11–13), each member of the family carries the same *ELANE* mutation site and exhibits completely different clinical phenotypes, indicating that the complex relationship between the *ELANE* gene and clinical phenotype needs further research. 3) Genotype and poor prognosis: the high-frequency mutation sites of poor prognosis were A57V, L121H, L121P, c.597 + 1G>A, c.597 + 1G>T, S126L, C151Y, C151S, G214R, and C223X. All 10 mutation sites reported cases of acute myeloid leukemia. The related sites of myelodysplastic syndrome were L121P, c.597 + 1G>T, S126L, C151Y, C151S, and G214R, and the related sites of acute lymphoblastic leukemia were A57V and G214R (5, 14, 15).

Prevention of infection is very important for SCN and CyN with *ELANE* mutations (24), especially for umbilical and perianal care in newborns and infants. G-CSF is the first-line treatment to improve clinical symptoms at present (7), and hematopoietic stem cell transplantation is an important radical treatment (2, 25). The Severe Chronic Neutropenia International Registry (SCNIR) recommends an initial treatment dose of 5.00 μg/(kg·d), with an absolute neutrophil count exceeding based on the patient’s clinical symptoms and neutrophil levels (14). The treatment target was above 1.00–1.50 × 10^9^/L, and the dosage of G-CSF <25.00 μg/(kg·d) can significantly improve the clinical symptoms of most patients (26, 27). According to this study, SCN patients mostly use 5.00 μg/(kg·d) as the initial dose, with a distribution trend of 6.00 ± 4.00 μg/(kg·d), while CyN patients mostly choose 2.00 μg/(kg·d) as the initial dose, with a distribution trend of 2.00 ± 3.17 μg/(kg·d). Of G-CSF refractory patients, 39.47% were associated with severe outcomes, with statistical differences (p < 0.05).

Regarding the transplantation guidelines for *ELANE* mutation patients, the following points were summarized. i) The treatment effect of G-CSF is poor: 1) starting to use G-CSF at the age of <1 year, the cumulative dose gradually increases with age to control the condition (14, 27). 2) The number of neutrophils cannot increase to 0.5 × 10^9^/L or continues to decrease (27). 3) There are still recurrent or severe infections after the use of G-CSF (1). 4) The neutrophils of G-CSF refractory patients do not increase after high-dose G-CSF >5 μg/(kg·d) treatment (2, 14). ii) During the follow-up process, mutations in the *CSF3R* gene were found (5, 27). iii) There was a risk of malignant transformation to MDS/AML/ALL (1, 2, 5, 26).

According to this study, patients receiving HSCT carry *ELANE* hotspot mutations G214R, S126L, P139L, and c.597 + 1G>A. The average age of transplantation is 5.65 years; 77.00% of donors are unrelated donors, and the grafts are cord blood stem cells (40.00%), bone marrow hematopoietic stem cells (36.00%), and peripheral hematopoietic stem cells (24.00%). The most common pre-treatment regimens are Bu-Cy-ATG (31.03%) and Flu-Bu-ATG (12.07%). The treatment methods for this disease are limited. How to better apply G-CSF and HSCT treatment methods and seek more treatment methods, such as NE inhibitors as alternative treatment drugs for G-CSF and gene editing, is the future research direction (8, 28).

This study explores the clinical characteristics, gene mutations, genotypes and phenotypes, and treatment methods of *ELANE* gene-related SCN and CyN. The complex relationship between the *ELANE* gene and clinical phenotype, and the mechanism by which high-dose G-CSF leads to poor prognosis, is not fully understood. Hence, further research is needed.

## Data availability statement

The original contributions presented in the study are included in the article/supplementary materials, further inquiries can be directed to the corresponding author/s.

## Author contributions

YX: Data curation, Formal Analysis, Investigation, Validation, Writing – original draft, Writing – review & editing. NW: Data curation, Supervision, Validation, Writing – review & editing. XJ: Supervision, Validation, Writing – review & editing. AL: Supervision, Validation, Writing – review & editing. ZZ: Conceptualization, Project administration, Supervision, Validation, Writing – review & editing.

## References

[1] Rotulo GA Plat G Beaupain B Blanche S Moushous D Sicre de Fontbrune F . Recurrent bacterial infections, but not fungal infections, characterise patients with ELANE-related neutropenia a French Severe Chronic Neutropenia Registry study. Br J Haematol. (2021) 194:908–920 2021. doi: 10.1111/bjh.17695 34340247

[2] Rotulo GA Beaupain B Rialland F Paillard C Nachit O Galambrun C . HSCT may lower leukemia risk in ELANE neutropenia: a before-after study from the French Severe Congenital Neutropenia Registry. Bone marrow Transplant. (2020) 55:1614–22. doi: 10.1038/s41409-020-0800-1 PMC7091645 31992846

[3] Gilman PA Jackson DP Guild HG . Congenital agranulocytosis: prolonged survival and terminal acute leukemia. Blood. (1970) 36:576–85. doi: 10.1182/blood.V36.5.576.576 4319697

[4] Rydzynska Z Pawlik B Krzyzanowski D Mlynarski W Madzio J . Neutrophil elastase defects in congenital neutropenia. Front Immunol. (2021) 12:653932. doi: 10.3389/fimmu.2021.653932 33968054 PMC8100030

[5] Makaryan V Zeidler C Bolyard AA Skokowa J Rodger E Kelley ML . The diversity of mutations and clinical outcomes for ELANE-associated neutropenia. Curr Opin Hematol. (2015) 22:3–11. doi: 10.1097/MOH.0000000000000105 25427142 PMC4380169

[6] Ancliff PJ . Congenital neutropenia. Blood Rev. (2003) 17:209–16. doi: 10.1016/S0268-960X(03)00019-5 14556775

[7] Hashem H Abu-Arja R Auletta JJ Rangarajan HG Varga E Rose MJ . Successful second hematopoietic cell transplantation in severe congenital neutropenia. Pediatr Transplant. (2018) 22(1):e13078. doi: 10.1111/petr.13078 29076228

[8] Rao S Yao Y Soares de Brito J Yao Q Shen AH Watkinson RE . Dissecting ELANE neutropenia pathogenicity by human HSC gene editing. Cell Stem Cell. (2021) 28:833–45.e5. doi: 10.1016/j.stem.2020.12.015 33513358 PMC8106646

[9] Klimiankou M Mellor-Heineke S Klimenkova O Reinel E Uenalan M Kandabarau S . Two cases of cyclic neutropenia with acquired CSF3R mutations, with 1 developing AML. Blood. (2016) 127:2638–41. doi: 10.1182/blood-2015-12-685784 27030388

[10] Setty BA Yeager ND Bajwa RP . Heterozygous M1V variant of ELA-2 gene mutation associated with G-CSF refractory severe congenital neutropenia. Pediatr Blood Cancer. (2011) 57:514–5. doi: 10.1002/pbc.23018 21618407

[11] Boxer LA Stein S Buckley D Bolyard AA Dale DC . Strong evidence for autosomal dominant inheritance of severe congenital neutropenia associated with ELA2 mutations. J Pediatr. (2006) 148:633–6. doi: 10.1016/j.jpeds.2005.12.029 16737875

[12] Newburger PE Pindyck TN Zhu Z Bolyard AA Aprikyan AA Dale DC . Cyclic neutropenia and severe congenital neutropenia in patients with a shared ELANE mutation and paternal haplotype: Evidence for phenotype determination by modifying genes. Pediatr Blood Cancer. (2010) 55:314–7. doi: 10.1002/pbc.22537 PMC2913300 20582973

[13] Cho HK Jeon IS . Different clinical phenotypes in familial severe congenital neutropenia cases with same mutation of the ELANE gene. J Korean Med Sci. (2014) 29:452–5. doi: 10.3346/jkms.2014.29.3.452 PMC3945145 24616599

[14] Skokowa J Dale DC Touw IP Zeidler C Welte K . Severe congenital neutropenias. Nat Rev Dis Primers. (2017) 3:17032. doi: 10.1038/nrdp.2017.32 28593997 PMC5821468

[15] Rosenberg PS Zeidler C Bolyard AA Alter BP Bonilla MA Boxer LA . Stable long-term risk of leukaemia in patients with severe congenital neutropenia maintained on G-CSF therapy: Short report. Br J Haematology. (2010) 150:196–9. doi: 10.1111/j.1365-2141.2010.08216.x PMC2906693 20456363

[16] Benson KF Luty J Hadavi V Kariminejad R Kariminejad MH . Double *de novo* mutations of ELA2 in cyclic and severe congenital neutropenia. Hum Mutat. (2007) 28:874–81. doi: 10.1002/(ISSN)1098-1004 17436313

[17] Rezaei N Moin M Pourpak Z Ramyar A Izadyar M Chavoshzadeh Z . The clinical, immunohematological, and molecular study of Iranian patients with severe congenital neutropenia. J Clin Immunol. (2007) 27:525–33. doi: 10.1007/s10875-007-9106-y 17587155

[18] Nustede R Klimiankou M Klimenkova O Kuznetsova I Zeidler C Welte K . ELANE mutant-specific activation of different UPR pathways in congenital neutropenia. Br J haematology. (2016) 172:219–27. doi: 10.1111/bjh.13823 26567890

[19] Dinand V Yadav SP Bellanne´-Chantelot C Jain S Bhargava M Sachdeva A . Hepatic hemangioendothelioma in an infant with severe congenital neutropenia. Pediatr Hematol Oncol. (2012) 34:298–300. doi: 10.1097/MPH.0b013e318249a4dc 22510773

[20] Carlsson G Aprikyan AA Ericson KG Stein S Makaryan V Dale DC . Neutrophil elastase and granulocyte colony-stimulating factor receptor mutation analyses and leukemia evolution in severe congenital neutropenia patients belonging to the original Kostmann family in northern Sweden. Haematologica. (2006) 91:589–95. doi: 10.1097/MPH.0b013e318249a4dc 16670064

[21] Germeshausen M Deerberg S Peter Y Reimer C Kratz CP Ballmaier M . The spectrum of ELANE mutations and their implications in severe congenital and cyclic neutropenia. Hum Mutat. (2013) 34:905–14. doi: 10.1002/humu.2013.34.issue-6 23463630

[22] Kurnikova M Maschan M Dinova E Shagina I Finogenova N Mamedova E . Four novel ELANE mutations in patients with congenital neutropenia. Pediatr Blood Cancer. (2011) 57:332–5. doi: 10.1002/pbc.23104 21425445

[23] Shen X Song S Li C Zhang J . Synonymous mutations in representative yeast genes are mostly strongly non-neutral. Nature. (2022) 606:725–31. doi: 10.1038/s41586-022-04823-w PMC9650438 35676473

[24] Wang J Zhang H Wang Y Liang L Yang Z . Severe congenital neutropenia caused by ELANE gene mutation: A case report and literature review. Medicine. (2022) 101:e31357. doi: 10.1097/MD.0000000000031357 36343040 PMC9646559

[25] Carlsson G Fasth A Berglof E Lagerstedt-Robinson K Nordenskjöld M Palmblad J . Incidence of severe congenital neutropenia in Sweden and risk of evolution to myelodysplastic syndrome/leukaemia. Br J haematology. (2012) 158:363–9. doi: 10.1111/j.1365-2141.2012.09171.x 22624626

[26] Shu Z Li XH Bai XM Lagerstedt-Robinson K Nordenskjöld M Palmblad J . Clinical characteristics of severe congenital neutropenia caused by novel ELANE gene mutations. Pediatr Infect Dis J. (2015) 34:203–7. doi: 10.1097/INF.0000000000000522 25162927

[27] Rosenberg PS Alter BP Bolyard AA Bonilla MA Boxer LA Cham B . The incidence of leukemia and mortality from sepsis in patients with severe congenital neutropenia receiving long-term G-CSF therapy. Pediatr Infect Dis J. (2006) 107(12):4628–35. doi: 10.1182/blood-2005-11-4370 PMC1895804 16497969

[28] Makaryan V Kelley ML Fletcher B Bonilla MA Boxer LA Cham B . Elastase inhibitors as potential therapies for ELANE-associated neutropenia. J leukocyte Biol. (2017) 102:1143–51. doi: 10.1189/jlb.5A1016-445R PMC5597518 28754797

